# Major Issues and Challenges of Influenza Pandemic Preparedness in Developing Countries

**DOI:** 10.3201/eid1406.070839

**Published:** 2008-06

**Authors:** Hitoshi Oshitani, Taro Kamigaki, Akira Suzuki

**Affiliations:** * Tohoku University Graduate School of Medicine, Sendai, Japan

**Keywords:** influenza, pandemic, avian influenza, mitigation, developing country, vaccine, antiviral, perspective

## Abstract

Better preparedness for an influenza pandemic mitigates its impact. Many countries have started developing and implementing national influenza pandemic preparedness plans. However, the level of preparedness varies among countries. Developing countries encounter unique and difficult issues and challenges in preparing for a pandemic. Deaths attributable to an influenza pandemic could be substantially higher in developing countries than in industrialized countries. Pharmaceutical interventions such as vaccines and antiviral agents are less likely to be available in developing countries. The public health and clinical infrastructure of developing countries are often inadequate to deal with a widespread health crisis such as an influenza pandemic. Such an event will inevitably have a global effect. Therefore, improving pandemic preparedness in every country, particularly developing ones, is urgently needed.

## Abstract 2

Summary line: A pandemic is a global issue, and pandemic preparedness should be considered from a global perspective**.**

Avian influenza, caused by influenza A virus (H5N1), continues to cause outbreaks among poultry and wild birds worldwide. It has spread from Asia to other regions, including Europe, the Middle East, and Africa. The number of cases of human subtype H5N1 infection also continues to rise. These historically unprecedented outbreaks have raised serious global concerns about the imminent arrival of an influenza pandemic. The World Health Organization (WHO) urges countries to develop and implement national pandemic preparedness plans to mitigate the health and social effects of a pandemic (*1*). However, the level of preparedness varies among countries. In general, developing countries have limited financial and technical resources to strengthen pandemic preparedness. They also face some unique and difficult issues, which make preparing for a pandemic more challenging. These have not been addressed adequately during planning. Effective and feasible strategies are needed to mitigate the impact of the next influenza pandemic in developing countries.

## Major Issues

### Potential Impact of Next Influenza Pandemic in Developing Countries

When an influenza pandemic emerges, all countries worldwide will inevitably be affected. However, the impact may vary both between and within countries. The estimated deaths for various countries during the Spanish flu pandemic from 1918 to 1920 shows that mortality rates in Europe and North America were significantly lower than those in Asia, Sub-Saharan Africa, and Latin America (*2*,*3*). A recent study that estimated the global impact of the Spanish flu pandemic indicated that a considerable difference in mortality rates was observed between high- and low-income countries (*4*). Why the pandemic caused such high mortality rates in developing countries is not entirely clear. Several factors may have been involved, including lack of access to adequate medical care, weak public health infrastructures, social factors such as housing conditions and population density, and host factors such as nutritional status and co-existing medical conditions. Another potential factor likely to influence mortality in a future pandemic is the high HIV/AIDS prevalence in some developing countries. Excess deaths attributed to pneumonia or influenza are significantly higher in HIV-positive persons during influenza seasons (*5*). HIV co-infection with a pandemic virus can be associated with more severe infections, which may further raise death rates in countries with high HIV/AIDS prevalence.

For these reasons, deaths associated with a future pandemic may be greater in developing countries than in industrialized countries. One study concluded that 96% of the estimated 62 million deaths in a future pandemic would occur in developing countries (*4*). The impact of such high mortality rates obviously needs to be taken into account when creating pandemic preparedness plans for developing countries. However, no appropriate model that can estimate the impact of an influenza pandemic in developing countries exists. Models are based on data from industrialized countries (*6*), which may underestimate the actual impact of a pandemic in developing countries.

### Availability of Vaccines and Antiviral Agents in Developing Countries

Several possible interventions can be implemented to control or mitigate the effects of an influenza pandemic, which include pharmaceutical interventions such as vaccines and antiviral agents, and nonpharmaceutical interventions such as quarantine, isolation, social distancing, and personal hygiene (*7*). Pharmaceutical interventions are needed for mitigating the impact of an influenza pandemic (*8*). Vaccines for subtype H5N1 viruses are currently being developed, and clinical trials are under way (*9*,*10*). However, worldwide vaccine production capacity is limited and is primarily in industrialized countries, where most seasonal influenza vaccine is produced (*11*). A recent WHO report estimated that the worldwide vaccine production capacity for current influenza vaccines is 350 million doses per year (*12*). That level of production is clearly insufficient to supply vaccines to all countries. Only a limited number of vaccine doses would be available, particularly in the early stages of the pandemic, and most of them would likely be supplied to industrialized countries. Many countries, especially developing countries, will be forced to confront the next pandemic with few or no available vaccines.

Antiviral agents are also considered effective for an influenza pandemic. They are particularly useful in the early stages of a pandemic when there is a shortage of vaccines (*13*). Two groups of antiviral agents for influenza are currently available, including M2 ion-channel inhibitors (amantadine and rimantadine) and neuraminidase inhibitors (oseltamivir and zanamivir). Neuraminidase inhibitors are preferred because some influenza viruses show high frequencies of resistance to M2 ion-channel inhibitors (*14*). Stockpiling of neuraminidase inhibitors is under way in many industrialized countries as part of national influenza pandemic preparedness (*15*). However, the stockpiles of antiviral agents available in developing countries are small and limited. WHO has global and regional stockpiles of antiviral agents, which are limited and are specifically used for early response and containment. The stockpile of antiviral agents is insufficient for a global pandemic.

The most critical limiting factor for stockpiling of neuraminidase inhibitors in developing countries is their high cost. One treatment course of oseltamivir (i.e., 10 tablets) costs US $15, even at a discount rate (*16*), which is far too expensive for developing countries. Some industrialized countries have set a target to stockpile oseltamivir to treat 25% of the general population. To purchase adequate oseltamivir for 25% of the total population, only 0.11% of the total annual health expenditure is required in high-income countries. In low-income countries, however, the expense would be 12.9% of the annual expenditure (Table 1). Therefore, it is not feasible for low-income countries to allocate scarce resources to stockpile sufficient quantities of oseltamivir for an unpredictable influenza pandemic.

**Table 1:** Cost of purchasing oseltamivir to cover 25% of population with regard to total health expenditure in countries with different economic status*

Category of country	Average GNP, per capita†	Average annual health expenditure, per capita†	Cost of 1 treatment course of oseltamivir, % annual health expenditure
High income	30,168	3,376	0.11
Upper middle income	4,310	280	1.34
Lower middle income	1,364	77	4.87
Low income	753	29	12.93

*Data obtained from World Health Organization website (www.who.int/nha). GNP, gross national product. †In US$.

### Limitations of Pharmaceutical Interventions

The recent efforts to increase global availability of vaccines and antiviral agents can contribute to increasing the global availability of these pharmaceutical interventions. However, increased availability alone will not solve all the problems in many countries. Several other issues need to be addressed to implement pharmaceutical interventions. These pharmaceutical commodities, including syringes and needles for vaccines, should be delivered to healthcare facilities throughout the country. That is a difficult logistic challenge for many developing countries. Human resources are also required to implement these interventions. Yet, there are some uncertainties about the effectiveness of these pharmaceutical interventions. Even neuraminidase inhibitors may not be fully effective for a pandemic virus, whose pathogenesis in human hosts differs from that of seasonal influenza viruses. Another potential problem with the antiviral drugs is the risk that resistant strains will emerge. Vaccines may not be effective because of antigenic differences between a vaccine strain and a pandemic virus, or for other reasons. Full-scale implementation of pharmaceutical interventions that requires enormous financial and human resources may not be the best use of limited resources in developing countries. The governments, international organizations such as WHO, and donors should consider various factors when providing support for pharmaceutical interventions in developing countries. Maintaining a balance between pharmaceutical and nonpharmaceutical interventions is necessary to achieve the best use of limited resources.

### Lack of Medical and Public Health Infrastructure to Cope with an Influenza Pandemic

During an influenza pandemic, morbidity and mortality may be extremely high. Healthcare facilities would be quickly overwhelmed with increased numbers of patients. In the United States alone, an estimated 18–42 million outpatient visits and 314,000–734,000 hospitalizations could occur (*6*). The surge capacity in healthcare systems will likely be insufficient to cope with this rise in patient numbers, even in industrialized countries (*17*,*18*). Healthcare resources such as the number of physicians, nurses, and available hospital beds are limited in developing countries. In some countries, resources are insufficient to cope with patients even during normal circumstances. Hospitals and clinics in developing countries will be easily overwhelmed by the increasing number of patients during an influenza pandemic.

Using the method described by Wilson et al. (*19*), we estimated the number of required hospital admissions for countries of varying economic status. The percentages of available hospital beds occupied by influenza patients at incidence rates of 15% and 35% were calculated by using FluSurge software, version 2.0 (*20*). Demographic data were obtained from the US Census Bureau website (www.census.gov/ipc/www/idb) and information related to the number of available beds was obtained from a WHO database (WHOSIS, www.who.int/whosis/en). Results are shown in Table 2. The percentage of hospital beds required for patients with pandemic influenza is much higher in low-income countries than in high-income countries. With an incidence rate of 35%, up to 79.1% of hospital beds are required for patients with pandemic influenza in low-income countries. In countries like Bangladesh and Nepal, >100% of beds would be required for patients with pandemic influenza, even at the incidence rate of 15% (data not shown). This model is based on data from the United States, and the difference in disease severity among the countries was not considered. This model may underestimate the hospital bed requirements in developing countries, where a pandemic virus may cause more severe infections. Some hospitalized patients will require mechanical ventilation (*17*), but few mechanical ventilators, if any, are available in many hospitals in developing countries.

**Table 2:** Hospital bed requirements during an influenza pandemic in countries with different economic status*

Category of country	Mean no. hospital beds/1,000 population (range)	Mean no. hospital beds required, as % of available hospital beds (range)	
		15% Incidence rate	35% Incidence rate
High income (N = 38 per capita)	50.7 (21–196)	8.9 (2.2–15.5)	20.8 (5.2–35.7)
Upper middle income (N = 28 per capita)	45.1 (9–99)	10.6 (3.9–30.1)	24.8 (9.0–70.3)
Lower middle income (N = 46 per capita)	30.0 (5–112)	15.5 (2.4–50.0)	36.2 (5.7–116)
Low income (N = 19 per capita)	26.2 (1.5–132)	33.9 (2.5–164)	79.1 (5.9–383)

*Only those countries with data on hospital beds and that were included in the World Bank country classification were included in the analyses. African countries are not incorporated in the analyses because they have no hospital bed data.

During an influenza pandemic, additional essential medical supplies such as gloves, masks, syringes, antipyretics, and antimicrobial agents will also be required. These supplies are insufficient in healthcare facilities in developing countries, even in nonemergency situations. Lack of these supplies may hamper provision of adequate medical care for patients with pandemic influenza. Basic personal protective equipment such as disposable gloves and surgical masks are needed for protecting healthcare workers. Antimicrobial agents are expected to be effective for secondary bacterial pneumonia, which can be a major cause of death for patients with pandemic influenza (*21*). Therefore, proper treatment with antimicrobial agents can be crucial for preventing deaths. However, in some developing countries, sufficient stocks of essential drugs, including antimicrobial agents, are often unavailable.

In countries with limited healthcare resources, providing routine medical care for other conditions may become difficult during a pandemic. For example, the treatment for tuberculosis or the antiretroviral treatment for AIDS patients may not be provided because of disruption in healthcare systems. Maintaining other public health programs, such as vaccination, may also be difficult when most of public health resources are spent for the response to a pandemic.

## Future Directions

### Improving Planning Process

To minimize the impact of an influenza pandemic, good preparedness plans need to be developed. With the increasing risk for a pandemic caused by the spread of influenza A virus (H5N1), most countries have started such planning. These national plans were recently reviewed from different perspectives (*15*,*22*–*24*). The level of planning in many developing countries is still inadequate to deal with such a major public health crisis. Some plans are based on the available plans of industrialized countries, or follow similar approaches to those of industrialized countries. As described above, the approaches used by industrialized countries may not be feasible or appropriate for developing countries. In addition, each country has specific issues, and therefore it should develop a plan based on its own requirements. This task can be difficult for most developing countries because they have little or no expertise with influenza and pandemic preparedness. For the few infectious disease experts working on infectious diseases in each country, numerous competing priorities exist, such as HIV/AIDS, malaria, tuberculosis, and vaccine-preventable diseases. Feasible, user-friendly tools are needed to assist these countries. WHO has developed several such tools, including a checklist for national preparedness (*25*). However, these tools describe the general approaches to pandemic preparedness and are not specifically designed for countries with limited resources. For developing countries more practical tools are needed, among them models to estimate the impact of a pandemic in developing countries, a list of feasible interventions to mitigate the impact of pandemic without available pharmaceutical interventions, and planning guidelines for hospitals with limited resources.

### Increasing Availability of Antiviral Agents and Vaccines

If the next pandemic occurs in a few years, vaccines and antiviral agents, particularly neuraminidase inhibitors, may not be available as a main intervention in developing countries. Availability needs to be increased to fill the gaps between developed and industrialized countries. WHO recommends an increase in worldwide vaccine production to meet the demand during a pandemic (*12*). Several countries have initiated projects to improve influenza vaccine production with technical and financial support from WHO and donors. However, improved vaccine production capacity is not sustainable if only used for pandemic influenza vaccines. The use of seasonal influenza vaccines would also need to increase in these countries. However, the cost of the vaccines (US $3–$7 per dose) is a barrier in increasing their use (*12*). There is also little available evidence on the effectiveness and cost benefits of seasonal influenza vaccines in tropical developing countries. Further efforts should be made to reduce the cost and to collect additional scientific data to increase the use of seasonal influenza vaccines.

Some approaches have been proposed and tested to reduce the amount of antigens per vaccine dose for pandemic vaccine so that more vaccines, including adjuvant and whole virion vaccines, can be supplied (*10*). The world is expected to have an increased capacity to produce vaccines for pandemic influenza viruses by 2010 (*12*). In some countries, the vaccines for the subtype with a pandemic potential are being produced and stockpiled as a prepandemic vaccine, which can be a useful tool to mitigate the impact of a pandemic (*26*). However, both pandemic and prepandemic vaccines would not be available in developing countries unless an international mechanism exists to share such vaccines with them at a low cost.

Some actions have also been taken to reduce the cost of neuraminidase inhibitors such as oseltamivir. It is being produced in sublicensing companies in developing countries to increase its supply at a lower cost. However, oseltamivir may still not be affordable for many developing countries. In industrialized countries, M2 ion-channel inhibitors are not considered a first choice of treatment because of the high rate of resistance to these inhibitors. However, amantadine is much cheaper than neuraminidase inhibitors and is more widely available. Most subtype H5N1 isolates that belong to clade 1 are resistant to amantadine, but many clade 2 viruses are still susceptible to amantadine (*27*). M2 ion-channel inhibitors can be a valid option for a pandemic, especially in developing countries (*28*). The value of M2 ion-channel inhibitors as a treatment option for an influenza pandemic should be evaluated further.

### Providing Better Medical Care

The health consequences of a pandemic, including deaths, can be substantially reduced by providing better medical care. Several issues need to be addressed to provide adequate medical care during a pandemic. First, essential medical supplies such as masks, gloves, and antimicrobial agents should be available in hospitals and clinics. The stockpiles of these basic supplies can be more cost-effective in developing countries than the stockpiles of more expensive antiviral agents. Guidelines on the types and quantity of essential items that are required in hospitals and clinics should be developed. Second, healthcare personnel should be trained for infection control measures. Even surgical masks are not commonly used in many developing countries, and hand hygiene practices are not always followed. Basic training on infection control should be provided to improve pandemic preparedness in healthcare settings. Third, healthcare and public health systems need to be maintained to minimize the impact of a pandemic. These systems should be maintained to deal not only with a pandemic but also with other health problems such as malaria, tuberculosis, and HIV.

### Developing Feasible Mitigation Strategies

More feasible and effective strategies should be developed as soon as possible to mitigate the negative impact of an influenza pandemic in developing countries. Since the availability of pharmaceutical interventions in developing countries is less likely, nonpharmaceutical interventions such as social distancing and personal hygiene may be the only available interventions. Public health measures such as school closure and household quarantine have been evaluated by using mathematical models for their effectiveness in mitigating the impact of a pandemic (*29*,*30*) and may have potential beneficial effects. However, the models suggest that substantial benefits of these measures require implementation with antiviral prophylaxis or vaccines (*29*,*30*). The evidence for effectiveness of public health measures is limited and is based primarily on experience in industrialized countries (*31*,*32*). For example, handwashing and hand hygiene have been highly publicized as a core management strategy for avian and pandemic influenza in developing countries (*33*). Although handwashing is effective in reducing the incidence of common diseases such as acute respiratory infections (*34*), data on its effectiveness specifically for community-acquired influenza infections are limited (*31*). Recommendations on nonpharmaceutical interventions have been based on available evidence (*35*). Accumulation of further scientific evidence for these measures, which can be implemented at a low cost, is urgently required.

### Strengthening Core Capacities

Many health programs in developing countries depend on financial support from donors. Influenza had little donor interest before the current avian influenza outbreaks. More donor funds are available for avian and pandemic influenza. These funds are often earmarked for specific activities. However, a more general approach is required to improve pandemic preparedness in developing countries. Improving pandemic preparedness without establishing a proper national program for seasonal influenza is unrealistic. For example, increasing the availability of pandemic vaccines without increasing the use of vaccines for seasonal influenza is difficult. It is also difficult to implement infection control measures in hospitals and personal hygiene during a pandemic if they are not routinely implemented for seasonal influenza and other infections.

Lack of adequate infrastructure and technical expertise is a fundamental issue for developing countries, not only for influenza pandemic preparedness but also for any other infectious disease threats. Revised International Health Regulations (2005) were adopted at the World Health Assembly in 2005, under which each country is required to have core capacities for disease surveillance and response (*36*). Strengthening the core capacity in each country should be an essential step to improve preparedness for any public health emergency, including an influenza pandemic. Although some actions should be taken immediately to address urgent issues regarding a pandemic threat posed by influenza A (H5N1), a long-term vision is required to establish such core capacity in every country.

### Strengthening International Collaboration

An influenza pandemic will spread to every corner of the world; hence, every country must be prepared for such a global event. All human cases of infection with influenza A virus (H5N1) have so far occurred in less industrialized countries, and thus the pandemic virus is likely to emerge from these countries. Epidemiologic models have indicated the possibility of rapid containment of the virus with a pandemic potential (*37*,*38*). WHO has stockpiles of oseltamivir specifically for the early containment of a potential pandemic. However, the window of opportunity is narrow, and early containment operations should be initiated as soon as the initial sign of a potential pandemic is detected. Timely sharing of the virus strains and relevant information is essential for such containment to be successful.

Sharing of the virus stains is also critical to develop pandemic vaccines. However, some countries do not share the virus strains with WHO reference laboratories. These countries argue that the virus strains from their countries would be used to develop pandemic vaccines that would only be available for rich countries (*39*). Developing countries have no incentives to share the virus strains if they do not benefit from the vaccines developed from these strains. The gaps in resources, including vaccine production capacity between the developing and industrialized countries, hinder the global effort to respond to a pandemic. Unequal distribution of resources, including antiviral stockpiles, could also be a major international issue when an influenza pandemic occurs. Countries with limited or no antiviral stockpiles and other resources may not be able to cope with the pandemic. A pandemic poses a serious threat to global health security if large gaps in capacity and available resources continue to persist. Large numbers of people may attempt to cross international borders to obtain better medical care, including antiviral treatment, or to escape a chaotic situation. Preparing for a pandemic by simply strengthening preparedness within a single country is not possible. A pandemic is a global issue, and pandemic preparedness should be considered from a global perspective.
